# Bacterial lipopolysaccharide induces apoptosis in the trout ovary

**DOI:** 10.1186/1477-7827-4-46

**Published:** 2006-08-31

**Authors:** Simon MacKenzie, Nuria Montserrat, Mario Mas, Laura Acerete, Lluis Tort, Aleksei Krasnov, Frederick W Goetz, Josep V Planas

**Affiliations:** 1 Departament de Fisiologia, Facultat de Biologia, Universitat de Barcelona, Barcelona, Spain; 2 Unitat de Fisiologia Animal, Departament de Biologia Cellular, Fisiologia i d'Immunologia, Facultat de Ciencies, Universitat Autonoma de Barcelona, Bellaterra, Barcelona, Spain; 3 Institute of Applied Biotechnology, University of Kuopio, Kuopio, Finland; 4 Great Lakes Water Institute, University of Wisconsin-Milwaukee, Wisconsin, USA; 5 Akvaforsk, PO Box 5010, N-1432 Ås, Norway

## Abstract

**Background:**

In mammals it is well known that infections can lead to alterations in reproductive function. As part of the innate immune response, a number of cytokines and other immune factors is produced during bacterial infection or after treatment with lipopolysaccharide (LPS) and acts on the reproductive system. In fish, LPS can also induce an innate immune response but little is known about the activation of the immune system by LPS on reproduction in fish. Therefore, we conducted studies to examine the in vivo and in vitro effects of lipopolysaccharide (LPS) on the reproductive function of sexually mature female trout.

**Methods:**

In saline- and LPS -injected brook trout, we measured the concentration of plasma steroids as well as the in vitro steroidogenic response (testosterone and 17alpha-hydroxyprogesterone) of ovarian follicles to luteinizing hormone (LH), the ability of 17alpha,20beta-dihydroxy-4-pregnen-3-one to induce germinal vesicle breakdown (GVBD) in vitro, and that of epinephrine to stimulate follicular contraction in vitro. We also examined the direct effects of LPS in vitro on steroid production, GVBD and contraction in brook trout ovarian follicles. The incidence of apoptosis was evaluated by TUNEL analysis. Furthermore, we examined the gene expression pattern in the ovary of saline- and LPS-injected rainbow trout by microarray analysis.

**Results:**

LPS treatment in vivo did not affect plasma testosterone concentration or the basal in vitro production of steroids, although a small but significant potentiation of the effects of LH on testosterone production in vitro was observed in ovarian follicles from LPS-treated fish. In addition, LPS increased the plasma concentration of cortisol. LPS treatment in vitro did not affect the basal or LH-stimulated steroid production in brook trout ovarian follicles. In addition, we did not observe any effects of LPS in vivo or in vitro on GVBD or follicular contraction. Therefore, LPS did not appear to impair ovarian steroid production, oocyte final maturation or follicular contraction under the present experimental conditions. Interestingly, LPS administration in vivo induced apoptosis in follicular cells, an observation that correlated with changes in the expression of genes involved in apoptosis, as evidenced by microarray analysis.

**Conclusion:**

These results indicate that female trout are particularly resistant to an acute administration of LPS in terms of ovarian hormone responsiveness. However, LPS caused a marked increase in apoptosis in follicular cells, suggesting that the trout ovary could be sensitive to the pro-apoptotic effects of LPS-induced inflammatory cytokines.

## Background

A substantial body of evidence indicates that reproductive competence can be compromised by external insults in the form of contaminants or pathogens [1-3]. In particular, pathogens stimulate the innate immune response which entails the recognition of pathogen-associated molecular patterns by immune cells of the myeloid lineage and their subsequent activation [4]. This leads to an inflammatory reaction typically characterized by the production of immune factors such as pro-inflammatory cytokines and chemokines [5]. In mammals, including humans, activation of the innate immune system caused by infection with Gram-negative bacteria has detrimental consequences for the function of the ovary as well as for fertility and embryonic survival [6-8]. The immune-mediated effects of Gram-negative bacteria are caused by the lipid A fraction of lipopolysaccharide (LPS), also known as endotoxin, which is a constituent of the bacterial cell wall that is constantly shed into the environment of the bacteria. In mammals, the induction of an immune response by the administration of LPS, commonly used to mimic a bacterial infection, results in alterations in ovarian function. LPS administration *in vivo *increases apoptosis in the ovary [6], reduces the ovarian steroidogenic response to gonadotropin stimulation [6,9,10] and impairs embryonic survival [7,11]. In contrast to mammals, little is known about the physiological consequences of an immune challenge by LPS in lower vertebrates. In general, lower vertebrates are known to be remarkably resistant to the toxic effects of LPS [12]. Recently, it has been postulated that their lower sensitivity to LPS could be related to differences in the repertoire of LPS-signaling receptors present [13]. Despite their lower sensitivity to LPS, there is good evidence to support the notion that fish respond to LPS by activating a typical innate immune response. For example, LPS administration in fish increases phagocytic activity of leukocytes and the activity and plasma concentration of lysozyme [14,15].

In the mammalian ovary, most of the detrimental effects of LPS are mediated by inflammatory cytokines, such as tumor necrosis factor α (TNFα) and interleukins 1α and 1β, which directly stimulate apoptotic cell death and inhibit steroid production [16-19]. These cytokines can be produced systemically by the cellular constituents of the innate immune system or locally by resident and/or infiltrating macrophages in the ovary. Activated ovarian macrophages also produce chemokines in response to TNFα, such as monocyte chemoattractant protein-1 [20], providing a possible mechanism whereby LPS administration increases the number of macrophages in the ovary [6]. In addition to their role in defense against pathogens, ovarian macrophages and their secretory products play an important role in ovarian function [21]. Cytokines such as TNFα are also essential regulators of ovarian growth and differentiation, as evidenced, for example, by their stimulatory effects on cell proliferation [22,23]. The contrasting and often contradictory reports on cytokine effects in the mammalian ovary reflect the complexity of the biological action of immune factors in contributing to ovarian homeostasis, which is further confounded by the fact that ovarian cells may represent an additional source of immune factors, such as TNFα [24]. Similarly, in fish, LPS acts directly on macrophages to stimulate the expression of typical pro-inflammatory cytokines, such as TNFα and IL-1β [25-27]. Furthermore, a recent high-throughput analysis of expressed genes in LPS-stimulated rainbow trout macrophages has identified a large number of other factors characteristic of activated macrophages [28]. Therefore, fish immune cells respond to LPS by producing a vast array of immune factors that are an essential part of the defense mechanism against pathogens. However, to date there is no information on the effects of LPS and the ensuing activation of the innate immune system on reproduction in fish. Fish species such as trout, reproduce only once annually and release thousands of oocytes at the time of ovulation that have developed and matured synchronously within the ovary. Thus, the entire cohort of ovarian follicles can be put at risk by the activation of the innate immune system as a result of a bacterial infection. Given the well-described modulatory effects of LPS and macrophage-derived factors on the function of the mammalian ovary (see above) and given the ability of fish to develop an innate immune response when exposed to LPS, the present study was undertaken to investigate the effects of activating the innate immune system by LPS on the function of the ovary in trout.

## Methods

### Animals

Brook trout (*Salvelinus fontinalis*) were purchased from a private hatchery (Grand Haven, MI) and maintained in tanks supplied with flow-through water at 12.5°C under natural photoperiod at the University of Notre Dame (Notre Dame, IN). Fish (300–400 g) were staged according to the position of the germinal vesicle (GV) in 8–10 oocytes that were cleared using a solution previously described [29]. Fish at the preovulatory stage (GV located approximately two-thirds the distance from the center to the periphery of the oocyte) were briefly anesthesized in 3-aminobenzoic acid ethyl ester (0.1 g/l of water; Sigma, St. Louis, MO) and injected intraperitoneally with either saline or *E. coli *lipopolysaccharide (LPS) (3 mg/kg weight) once a day over four consecutive days. In each experiment, five fish were injected with saline and five fish were injected with LPS. Twenty four hours after the last injection, fish were anesthesized as described above, blood samples were taken by caudal vein puncture and fish were sacrificed by spinal transection prior to the collection of the ovaries. The dissected ovaries were immediately used for the various *in vitro *assays and also processed for the TUNEL assay and routine histology.

Gene expression analyses were performed on female rainbow trout (*Oncorhynchus mykiss*) using a microarray platform previously validated for rainbow trout [30,31]. Adult fish at a preovulatory stage (250–300 g) were purchased from a commercial fish hatchery (Piscifactoria de Sant Privat, Girona, Spain) and were maintained in tanks supplied with flow-through water under natural conditions of light and temperature at the Universitat Autònoma de Barcelona (Spain). Fish were given a single intraperitoneal injection of either saline (n = 10) or LPS (n = 10; 6 mg/kg wet weight) as described above and 24 and 72 hours after the injection, fish were sacrificed (five fish from each group at each of the two time points), their ovaries removed, snap frozen in liquid nitrogen and kept at -80°C until they were processed for RNA purification (see below). For this particular study, the rainbow trout was selected as the experimental species primarily due to the availability of a cDNA microarray platform designed and validated for this species. Furthermore, the LPS treatment regime was chosen because it was previously shown to be effective in inducing the expression of immune genes [32].

### Hormones and reagents

Coho salmon LH (sLH) was a kind gift from Dr. Penny Swanson (National Marine Fisheries Service, Seattle, WA) [33] and was dissolved directly in incubation medium. *E. coli *LPS was purchased from Sigma and dissolved directly in saline or incubation medium.

### Ovarian tissue incubations

Immediately after dissection, preovulatory brook trout ovaries were placed in ice-cold Cortland's medium and individual ovarian follicles were separated from each ovary on ice, as previously described [34]. For *in vitro *steroid production experiments, intact preovulatory brook trout follicles (migrating GV) were incubated (10 follicles/well/3 ml) in ice-cold Cortland's medium containing 0.2% BSA (fraction V; Sigma), in the absence or presence of different test compounds for 18 h at 12°C in an air atmosphere with gentle shaking (100 rpm). At the termination of the incubation, the medium and ovarian tissue were removed and stored at -20°C and -80°C, respectively, until assayed. For the *in vitro *oocyte maturation assay (germinal vesicle breakdown (GVBD)), brook trout ovarian follicles with peripheral germinal vesicles (GVs) were selected. Briefly, ovarian follicles were incubated in Cortland's medium (10 follicles/well/3 ml) in the presence of 17α,20β-dihydroxy-4-pregnen-3-one (17,20β-P, 100 ng/ml) for 48 h at 12°C in 6-well plates with shaking. At the termination of the incubations, the follicles were cleared [29] and scored for the presence or absence (GVBD) of the GV as previously described [35]. For the follicle contraction experiments, punctured brook trout preovulatory follicles (migrating GV) were incubated in Cortland's medium (10 follicles/well/3 ml) in the presence of the test compounds for 8 hours at 12°C. Follicle contraction was determined by measuring the weight of the 10 follicles in each replicate and calculating the difference in follicle weight between the beginning and the end of the incubation period, as previously described and validated for epinephrine stimulation [36]. Since contraction results in the expulsion of yolk through the puncture site, decreases in follicle weight indicate increases in follicular contraction. In all the experiments described, statistical significance was determined by one-way ANOVA, followed by the Fisher's Protected Least Significant Difference test [37].

### Isolation of trout macrophages and production of macrophage conditioned medium

Brook trout macrophages were isolated from the head kidney and cultured as previously described [26]. To obtain supernatants from LPS-activated macrophages, macrophages were incubated at a density of 1 × 10^7 ^cells/ml in DMEM high glucose medium (Gibco) and stimulated in the absence or presence of LPS (10 μg/ml) for 12 h at 18°C under 5% CO_2_. This concentration of LPS has previously been shown to be effective in stimulating cytokine and chemokine expression in trout macrophages [26,32]. Following the incubation, the medium was collected and centrifuged for 10 min at 2000 × *g *at 4°C. Supernatants were pooled and used directly to incubate punctured brook trout preovulatory follicles in the follicle contraction experiments (see above).

### Steroid radioimmunoassays

The concentrations of testosterone, 17OH-P and 17β-estradiol in brook trout ovarian follicle incubates and plasma were measured directly using commercial radioimmunoassays (Schering-CIS, Madrid, Spain), as described previously [38,39]. The concentration of cortisol was determined by radioimmunoassay, as described previously [40], but with minor modifications. The antibody used for the cortisol radioimmunoassay was purchased from Biolink, S.L. (Costa Mesa, CA) and was used at a final dilution of 1:6000. The cross reactivity of this antibody with cortisol, 21-deoxycorticosterone, 11-deoxycortisol and 17β-hydroxyprogesterone was 100%, 11.4%, 8.9% and 1.6%, respectively.

### In situ TUNEL analyses

In order to determine the incidence of apoptosis in preovulatory ovaries from saline- and LPS-injected female brook trout, fragmentation of DNA in paraffin-embedded ovaries, previously de-yolked by gentle pressure, was detected by the terminal deoxynucleotidyl transferase (TdT)-mediated dUTP nick end labeling (TUNEL) technique [41]. Labeling of DNA strand breaks by TUNEL was performed using a commercial kit (In Situ Cell Death Detection Kit, Roche Diagnostics GmbH, Mannheim, Germany), according to the manufacturer's instructions. After labeling with fluorescein-dUTP, ovarian sections were visualized under a fluorescence microscope and digital images were captured from at least two sections of each ovary from saline-(n = 5) and LPS-injected (n = 5) fish. Negative (no TdT) and positive (DNase I treatment) controls were performed as indicated in the TUNEL kit using ovarian sections from LPS- and saline-injected trout and yielded the expected results (data not shown). Additional sections from de-yolked ovaries from saline- and LPS-injected brook trout were examined microscopically by routine hematoxylin-eosin staining.

### Microarray analyses

Microarray analyses were performed using a rainbow trout cDNA microarray platform previously validated and described [30,31,42] that has been deposited in GEO under accession number GPL1212. Briefly, total ovarian RNA was extracted from pooled ovaries from saline- (n = 5) and LPS-injected (n = 5) rainbow trout with TriReagent (Molecular Research Center, Cincinatti, OH) according to the manufacturer's specifications. Labeling with Cy3- and Cy5-dCTP (Amersham Pharmacia) was made using SuperScript III reverse transcriptase (Invitrogen) and oligo(dT) primer; cDNA was purified with Microcon YM30 (Millipore). The slides were pretreated with 1% BSA (fraction V), 5 × SSC, 0.1% SDS (30 min at 50°C) and washed with 2 × SSC (3 min) and 0.2 × SSC (3 min) and hybridized overnight in cocktail containing 1.3 × Denhardt's, 3 × SSC, 0.3% SDS, 2.1 μg/μl polyadenylate and 1 μg/μl yeast tRNA. All chemicals were from Sigma-Aldrich. Scanning was performed with ScanArray 5000 and images were processed with QuantArray (GSI Luminomics). The measurements in spots were filtered by criteria *I/B *≥ 3 and (*I-B*)/(*S*_*I *_+ *S*_*B*_) ≥ 0.6, where *I *and *B *are the mean signal and background intensities and *S*_*I*_, *S*_*B *_are the standard deviations. After subtraction of mean background, LOWESS normalization [43] was performed. To assess differential expression of genes, the normalized log intensity ratios (expression ratio) were analyzed with Student's t-test (P < 0.05).

## Results

### Effects of LPS on steroid production

**Figure 1:**
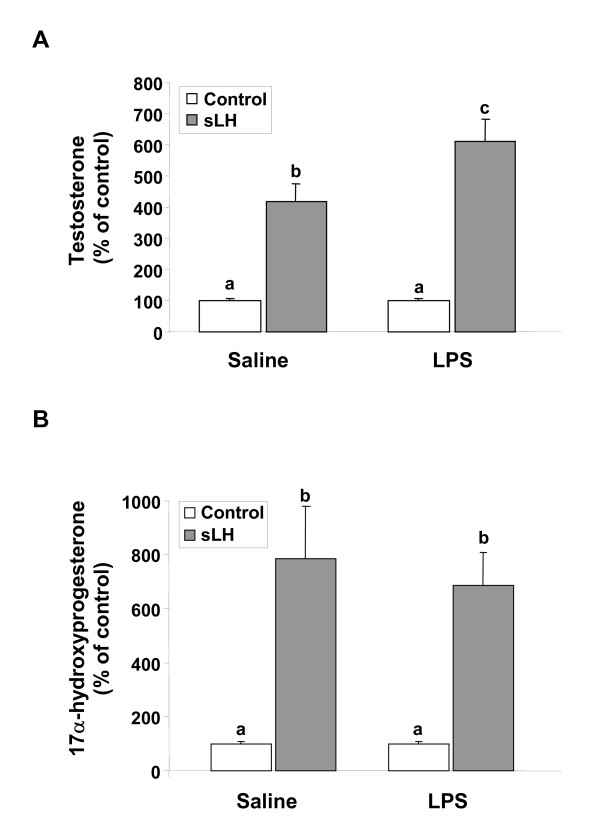
**Steroidogenic output of brook trout ovarian follicles treated withsaline and lipopolysaccharide (LPS) *in vivo***. Preovulatory trout follicles from saline- and LPS-treated fish were incubated for 18 h at 12°C in the absence or presence of salmon LH (sLH; 25 ng/ml). At the termination of the incubation period, testosterone (A) and 17α-hydroxyprogesterone (B) were measured in the medium. Each bar represents the mean ± SEM of five fish for each treatment, with each assayed in triplicate. The results are expressed as percent change with respect to the saline-injected control (no sLH) group which has been set at 100%. Statistically significant (p ≤ 0.05) differences among groups are indicated by different letters.

**Figure 2:**
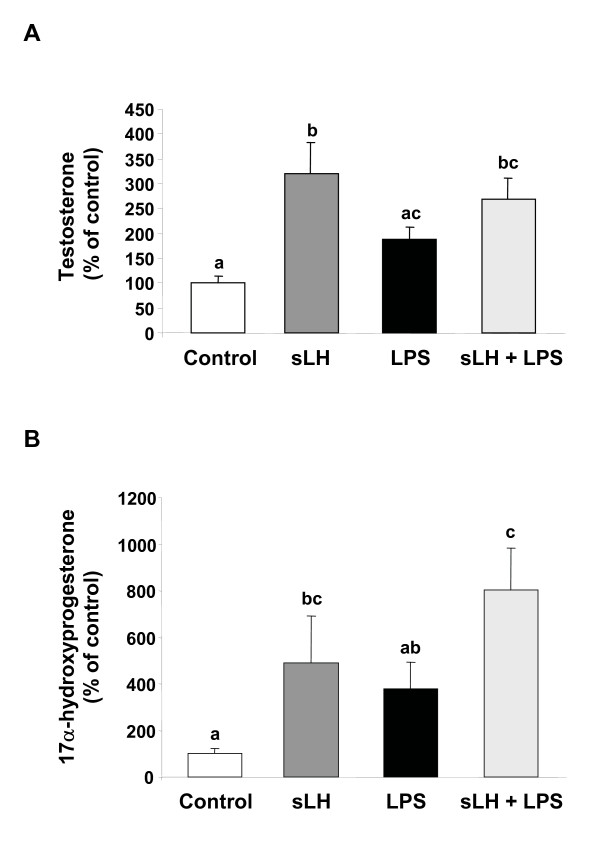
**Steroidogenic output of brook trout ovarian follicles treated with lipopolysaccharide (LPS) *in vitro***. Preovulatory trout follicles from untreated fish were incubated for 18 h at 12°C in the absence or presence of salmon LH (sLH; 25 ng/ml) and LPS (50 μg/ml). At the termination of the incubation period, testosterone (A) and 17α-hydroxyprogesterone (B) were measured in the medium. Each bar represents the mean ± SEM of three separate experiments, with each assayed in triplicate. The results are expressed as percent change with respect to the control group which has been set at 100%. Statistically significant (p ≤ 0.05) differences among groups are indicated by different letters.

The *in vivo *administration of LPS did not affect the basal *in vitro *production of testosterone (597.6 ± 131.2 pg/ml); however, LPS administration significantly potentiated the stimulatory effects of sLH on testosterone production by brook trout ovarian follicles (Fig. 1A). Neither the basal (65 ± 6.2 pg/ml) nor the sLH-stimulated production of 17OH-P appeared to be affected by the *in vivo *administration of LPS (Fig. 1B) and 17OH-P was produced at a much lower concentration than testosterone. When tested directly *in vitro*, LPS did not significantly affect the basal or the sLH-stimulated production of testosterone or 17OH-P by trout ovarian follicles (Fig. 2).

**Figure 3:**
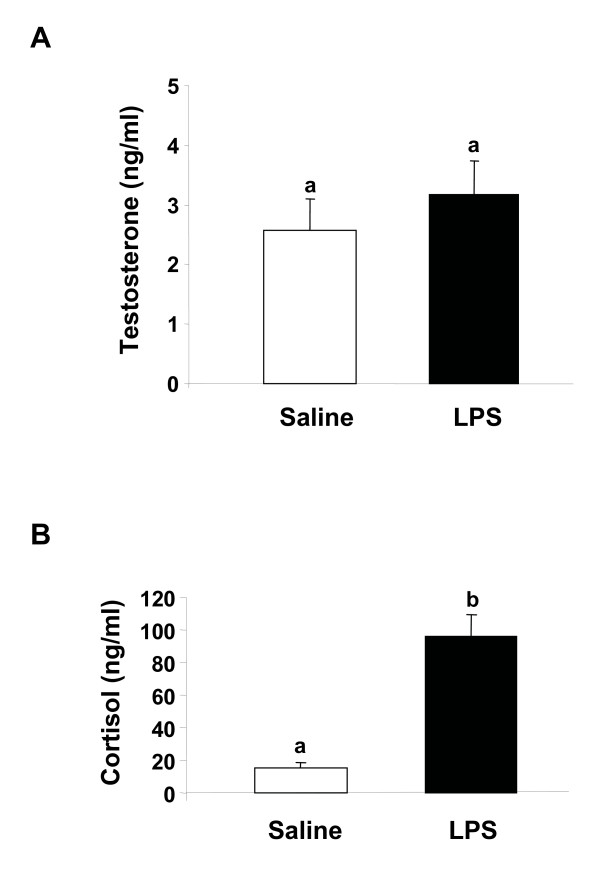
**Plasma steroid concentration in saline- and lipopolysaccharide (LPS)-treated brook trout females**. Plasma concentrations of testosterone (A) and cortisol (B) were measured in saline- and LPS-injected brook trout females. Each bar represents the mean ± SEM of five fish for each treatment, with each assayed in triplicate. Statistically significant (p ≤ 0.05) differences among groups are indicated by different letters.

In order to determine whether LPS administration *in viv*o affected the plasma concentration of steroids in female brook trout, we measured the concentration of testosterone, 17OH-P, 17β-estradiol and cortisol in plasma of saline- and LPS-injected female trout. Our results showed that testosterone plasma concentration was not affected by LPS administration (Fig. 3A). The plasma concentrations of 17OH-P and 17β-estradiol were not detectable (data not shown), in agreement with the known decrease in their circulating concentrations during the progression of final oocyte maturation and ovulation [44]. Interestingly, the plasma concentration of cortisol in LPS-treated female trout was 4–5 fold-higher than in saline-treated female trout (Fig. 3B).

### Effects of LPS on oocyte maturation

**Figure 4:**
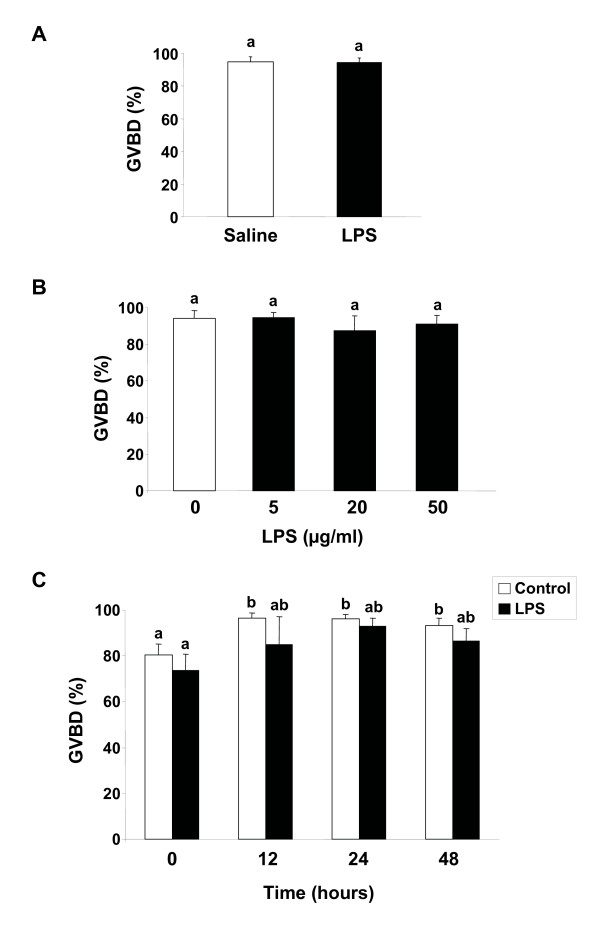
**Effects of lipopolysaccharide (LPS) on oocyte maturation in brook trout**. *A. Effects of in vivo LPS administration on brook trout oocyte maturation*. Preovulatory trout follicles from saline- and LPS-treated fish were incubated for 48 h at 12°C in the presence of 17α,20β-dihydroxy-4-pregnen-3-one (maturation-inducing steroid or MIS;100 ng/ml). At the termination of the incubation period, follicles were scored for the germinal vesicle breakdown (GVBD). Each bar represents the mean ± SEM of five fish for each treatment, with each assayed in triplicate. *B. Dose-response of LPS treatment in vitro on brook trout oocyte maturation*. Normal trout preovulatory ovarian follicles were incubated with MIS (100 ng/ml) and in the absence or presence of different concentrations of LPS (0–50 μg/ml) for 48 hours and scored for GVBD. The results show the mean ± SEM from three separate experiments, with each assayed in triplicate. *C. Time course of LPS treatment in vitro on brook trout oocyte maturation*. Normal trout preovulatory ovarian follicles were preincubated in the absence or presence of LPS (25 μg/ml) for different amounts of time (0–48 hours) and subsequently incubated in the presence of MIS (100 ng/ml), as indicated above, and assayed for GVBD. The results show the mean ± SEM from three separate experiments, with each assayed in triplicate. In all graphs, the results are expressed as percentage of total ovarian follicles at the peripheral GV stage that underwent GVBD. Statistically significant (p ≤ 0.05) differences among groups are indicated by different letters.

Ovarian follicles from saline- and LPS-injected brook trout showed no difference in their ability to undergo oocyte maturation in response to 17,20β-P *in vitro *(Fig. 4A). In addition, incubation of trout ovarian follicles with increasing concentrations of LPS (up to 50 μg/ml) or at various times (0 to 48 hours) prior to 17,20β-P stimulation did not affect their responsiveness to 17,20β-P *in vitro *(Figs. 4B,C).

### Effects of LPS on follicular contraction

**Figure 5:**
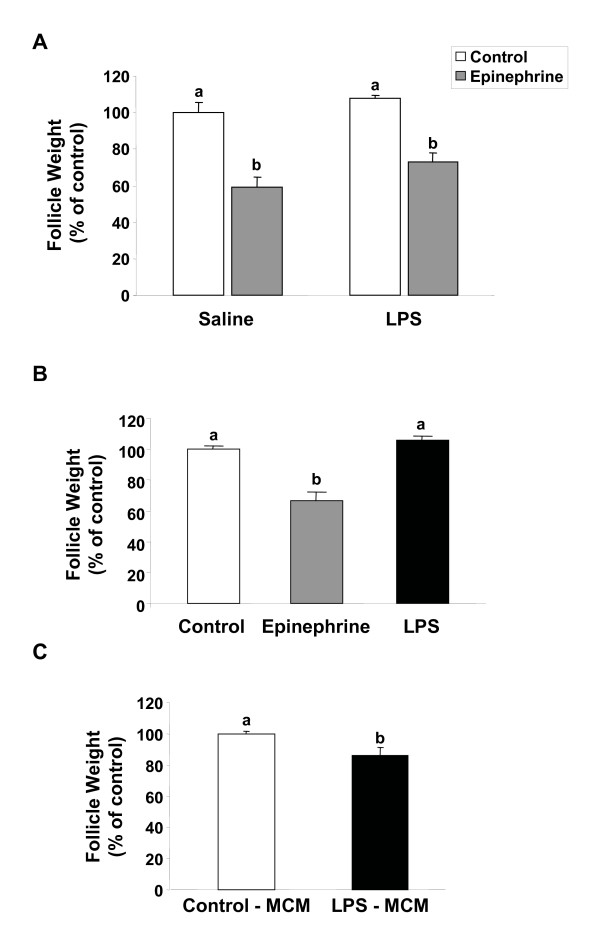
**Effects of lipopolysaccharide (LPS) on follicular contraction in brook trout**. *A. Effects of in vivo LPS administration on trout follicular contraction*. Punctured, preovulatory brook trout follicles from saline- and LPS-treated fish were incubated for 8 h at 12°C in the absence or presence of epinephrine (5μM). Each bar represents the mean ± SEM of five fish for each treatment, with each assayed in triplicate. The results are expressed as percent change with respect to the saline-injected control (no epinephrine) group which has been set at 100%. Statistically significant (p ≤ 0.0001) differences among groups are indicated by different letters. *B. Effects of LPS treatment in vitro on trout follicular contraction*. Punctured trout preovulatory ovarian follicles were incubated in the absence or presence of epinephrine (5μM) or LPS (25 μg/ml) for 8 hours. The results show the mean ± SEM from six separate experiments, with each assayed in triplicate. Statistically significant (p ≤ 0.001) differences among groups are indicated by different letters. *C. Effects of macrophage conditioned medium on trout follicular contraction*. Punctured, trout ovarian follicles were incubated with macrophage conditioned medium (Control-MCM) and with LPS-stimulated macrophage-conditioned medium (LPS-MCM) for 8 hours. Statistically significant (p < 0.05) differences among groups are indicated by different letters.

LPS administration *in vivo *(Fig. 5A) did not affect the basal rate of contraction of brook trout ovarian follicles or the contractile response to epinephrine, a well known stimulator of follicular contraction in the brook trout ovary [45]. In addition, LPS did not have any direct effects on the *in vitro *contraction of trout ovarian follicles (Fig. 5B). Interestingly, incubation of trout ovarian follicles in the presence of LPS-stimulated trout macrophage conditioned medium caused a small but significant (p < 0.05) increase in follicular contraction, as evidenced by the decrease in follicle weight (Fig. 5C).

### Effects of LPS on ovarian apoptosis

**Figure 6:**
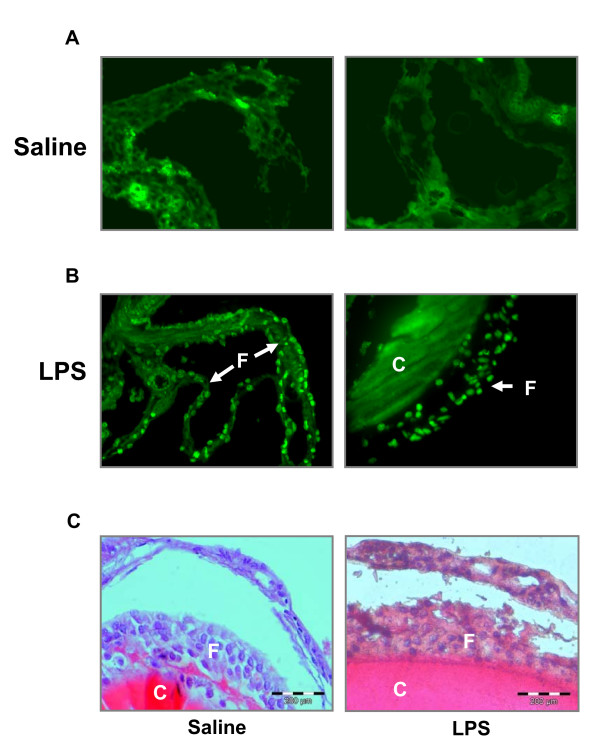
**Effects of lipopolysaccharide (LPS) administration on apoptosis in the brook trout ovary**. Sections of paraffin-embedded ovaries from saline (A)- and LPS (B)-injected brook trout were analyzed for *in situ *end-labeling by TUNEL. Two representative images of ovaries from saline- and LPS-injected trout are shown. In C, hematoxylin-eosin stained sections of representative ovaries from saline (left) and LPS-injected (right) brook trout are shown. Prior to fixation, the ovaries were de-yolked by gentle pressure. F, follicular cells; C, chorion.

Analysis of ovarian sections by *in situ *TUNEL indicated the presence of abundant positive nuclei indicative of apoptotic cells in the ovaries of LPS-injected female brook trout (Fig. 6B). In contrast, almost no positive nuclei were detected in ovaries of saline-injected female trout (Fig. 6A). In the ovaries of LPS-injected brook trout, apoptotic cells were found primarily in the granulosa and theca cell layers. Since oocytes were damaged by the process of de-yolking the ovarian follicles prior to fixation and histological processing, we could not evaluate the incidence of apoptosis directly within oocytes from LPS-injected female trout. In addition, no gross morphological differences were observed between the ovaries (de-yolked) of saline- and LPS-injected brook trout (Fig. 6C).

### Effects of LPS on ovarian gene expression

To investigate the effects of LPS-treatment on gene expression in the trout ovary, we used a trout cDNA microarray platform previously validated for studies involving response to stress and toxicity [30,31] and, more recently, to LPS stimulation [46] in trout.

In support of the observed increase in apoptosis in the ovary of LPS-treated fish, the expression of several genes known to be involved in apoptosis underwent changes in response to LPS administration in the ovary (Table 1). One the one hand, death-associated protein kinase 3, also refered to as ZIP kinase, was up-regulated at 24 hours post-injection. On the other hand, various genes involved in apoptosis were significantly down-regulated, such as beclin 1, apoptosis inhibitor 5 and cytochrome P450 2J2 at 72 hours post-injection, and cdk inhibitor p21 binding protein at 24 hours post-injection. In addition, the expression of telomerase was also reduced in the trout ovary in response to LPS administration at 72 hours post-injection. Also, the expression of the serum/glucocorticoid regulated kinase, an important mediator of cell survival signals [47], was similarly down-regulated at 72 hours after the LPS injection. Interestingly, the alpha 1 and alpha 2 chains of collagen type I were highly induced by LPS administration at 72 hours post-injection.

**Table 1:** Differentially expressed genes in the ovary of lipopolysaccharide (LPS)-treated female trout.

**Time (hours p.i.)**		
**24**	**72**	**Gene name**
**Up-regulated genes**		
1,57**	-1,16	Death-associated protein kinase 3 (ZIP-kinase).
1,32*	1,42	HLA class II histocompatibility antigen, gamma chain
1,56*	1,30	HLA class I histocompatibility antigen
1,25**	1,04	Myosin heavy chain, skeletal muscle, adult 1
1,33*	-1,15	Synapse associated protein 1
-1,07	1,56*	Collagen, type I, alpha 1chain
1,06	1,49*	Collagen, type I, alpha 2 chain
-1,05	1,39*	Phospholipase D family, member 4
1,05	1,56*	Microtubule-associated protein RP/EB
-1,14	1,28*	Translocon-associated protein, delta subunit precursor
**Down-regulated genes**		
-1,38*	1,09	Cdk inhibitor p21 binding protein
-1,35*	-1,23	Cytochrome oxidase subunit III-2
-1,39*	-1,15	Cytochrome oxidase subunit III-3
-1,49*	-1,12	Oxidoreductase UCPA
-1,22*	-1,02	Hypoxanthine-guanine phosphoribosyltransferase
-1,05	-1,32*	Beclin 1
1,23	-1,21*	Apoptosis inhibitor 5
1,04	-1,48*	Cytochrome P450 2J2
-1,01	-1,35*	Serum/glucocorticoid-regulated kinase
-1,14	-1,26**	Lactoferrin
-1,20	-1,49*	Alanine-glyoxylate aminotransferase
-1,07	-1,65*	Allograft inflammatory factor-1
-1,10	-1,31*	NADH dehydrogenase subunit 5–2
-1,06	-1,34*	Acyl-CoA dehydrogenase 9, mitochondrial
-1,05	-1,25*	High affinity immunoglobulin epsilon receptor alpha
-1,05	-1,24*	Troponin I, slow skeletal muscle
-1,18	-1,79*	Beta enolase
-1,05	-1,77*	Telomerase reverse transcriptase
-1,03	-1,23*	Coatomer epsilon subunit (Epsilon-COP).

The expression ratio (normalized log intensity ratio) data at 24 and 72 hours post-injection (p.i.) are shown. Significant differences between control and LPS-injected fish, as analyzed by Student's t-test, are indicated by asterisks (* p < 0.05; ** p < 0.01)

Several genes with an immune function appeared to be transcriptionally regulated in response to LPS in the trout ovary. For example, class I and II histocompatibility antigens were up-regulated in response to LPS administration at 24 hours. In addition, several immune-related genes were down-regulated in response to LPS administration only at 72 hours post-injection: immunoglobulin epsilon (IgE) receptor alpha subunit, allograft inflammatory factor-1, epsilon coat protein and lactoferrin.

The administration of LPS also caused a decrease in the expression of genes involved in metabolism, such as beta enolase, acyl-CoA dehydrogenase 9, alanine-glyoxylate aminotransferase, hypoxantine-guanine phosphoribosyl transferase and oxidoreductase (Table 1). Furthermore, the expression of the cytochrome c oxidase subunit III, part of the mitochondrial respiratory chain, was also decreased at 24 hours post-injection.

## Discussion

In mammals, bacterial LPS, commonly used to induce an immune response, is known to cause alterations in the normal function of the ovary. In the present study, we have sought to study for the first time the consequences of activating the innate immune system with LPS on ovarian function in fish. In particular, we examined the *in vivo *and *in vitro *effects of LPS administration on ovarian steroid production, oocyte maturation, follicular contraction, incidence of apoptosis and multiple gene expression in trout.

The results from the present study indicate that LPS administration increased apoptosis in the trout ovary. This conclusion is derived from the observed increase in the number of apoptotic cell nuclei in the follicular layers surrounding the trout oocyte and also from the observed changes in the ovarian expression of genes involved in the regulation of apoptosis. In particular, LPS administration increased the expression of ZIP-kinase, a positive mediator of apoptosis [48], in the trout ovary as early as 24 hours. Furthermore, LPS administration decreased the expression of several anti-apoptotic genes in the trout ovary: beclin 1, cdk inhibitor p21 binding protein, cytochrome P450 2J2, apoptosis inhibitor 5 and telomerase. For example, beclin 1 has been reported to have anti-apoptotic functions and to be involved in cell defense in mammals [49]. In addition, p21 is expressed in the murine ovary and is involved in follicular growth [50] and luteal differentiation and, more importantly, to have an anti-apoptotic function in granulosa cells [51]. Furthermore, cytochrome P450 2J2 produces epoxyeicosatrienoic acids which have anti-apoptotic properties [52], similar to apoptosis inhibitor 5 [53]. Finally, telomerase, a gene important for the life-span of cells due to its involvement in the synthesis and maintenance of telomeres, is known to have anti-apoptotic activity [54]. In addition to changes in genes involved in apoptosis, LPS administration caused a marked decrease in the expression of genes involved in metabolism and mitochondrial biogenesis. Similar changes were recently reported in rainbow trout macrophages stimulated with LPS [46] and, as in macrophages, one of the responses of the ovary to LPS administration could be to depress cellular metabolism and energy production by attenuating the expression of key genes involved in basic cellular functions. In the present study, we report on significant changes in gene expression that in most cases are less than two fold, supporting the notion that small expression differences, as those significantly detected using our validated microarray platform, could be functionally important.

The observed increase in apoptosis by LPS in the fish ovary suggests that the pro-apoptotic effects of LPS may be evolutionarily conserved since LPS induces ovarian apoptosis in mammals [6,55]. It is believed that the pro-apoptotic effects of LPS in the ovary of mammals and birds are mediated by pro-apoptotic cytokines. For instance, TNFα directly stimulates apoptosis in intact follicles and cultured granulosa cells [18,56,57]. Although the direct effects of cytokines on apoptosis in the fish ovary have not been examined to date, it is possible that the pro-apoptotic effects of LPS in the fish ovary could also be mediated by immune factors. In fact, trout macrophages increase the expression of cytokines in response to LPS [25,26,28,58]. Furthermore, the fish ovary contains receptors for cytokines. For example, a death-domain containing receptor of the TNF family and a TNF-decoy receptor are expressed in the zebrafish ovary [59] and in the granulosa cells of the trout ovary [60], respectively. At present it is not known if pro-apoptotic cytokines acting on fish ovarian cells could be produced systemically by LPS-activated immune cells in the pronephros, the hematopoietic organ in fish, or locally by resident ovarian macrophages. In the mammalian ovary, TNFα is expressed in various cellular compartments, including the oocyte, granulosa and theca cells, as well as in resident macrophages [21,24,61]. Furthermore, LPS administration *in vivo *increases the number of ovarian macrophages in the rat ovary [6], suggesting that infiltrating macrophages could represent a possible additional source of pro-apoptotic cytokines. In fish, pro-apoptotic cytokines are also produced in the ovary [59], although it remains to be shown if they are produced by macrophages and/or follicular cells. Interestingly, LPS induces the expression of a CCL4-like chemokine in the trout ovary [32], suggesting that trout immune cells, like their mammalian counterparts, could be recruited to the ovary in response to an immune challenge in the form of LPS. The regulated expression of several immune-related genes in the ovary of LPS-injected females strongly suggests that immune cells or factors are indeed found or produced by the ovary of the rainbow trout. For example, genes involved in antigen presentation, such as MHC class I and II major histocompatibility complex molecules, and shown to be expressed in LPS-stimulated trout macrophages [28], are also expressed in the rainbow trout ovary. Interestingly, mammalian luteal cells also express MHC class I and II molecules in response to immune stimuli [62]. In addition, it is possible that ovarian macrophages in trout could be induced by LPS to produce cytokines such as TNFα, as has been shown in trout macrophages differentiated *in vitro *[25,26]. Thus, once the necessary tools (antibodies, immune cells markers, etc.) become available, future studies can be conducted to determine the cellular localization of cytokine-expressing cells and their regulation by LPS, or to investigate the effects of LPS on macrophage influx into the trout ovary.

In marked contrast with the known inhibitory effects of *in vivo *LPS administration on ovarian steroidogenesis in mammals [6,9,10], LPS administration *in vivo *did not inhibit the production of ovarian steroids in trout. Our results indicate that the administration of high doses of LPS *in vivo *does not affect the circulating concentrations of sex steroids, the basal sex steroid output or the production of 17OH-P in response to gonadotropin stimulation in isolated trout ovarian follicles. However, the stimulatory effects of sLH on testosterone production were slightly potentiated in follicles from LPS-injected fish. The physiological significance of this result is not clear particularly in view of the lack of changes in the concentration of plasma testosterone in LPS-injected females. Moreover, trout ovarian follicles did not show a steroidogenic response to LPS *in vitro*. This is in contrast with the reported inhibitory effects of LPS on gonadotropin-stimulated steroid production in rat theca and granulosa cells [63,64], which are presumably attributed to the direct actions of LPS on steroidogenic cells [65].

Interestingly, the administration of LPS, either *in vitro *or *in vivo*, did not affect the ability of trout ovarian follicles to undergo oocyte maturation in response to 17,20β-P or to contract in response to epinephrine [29,36,45]. In contrast to the ineffectiveness of *in vivo *LPS administration on follicular contraction, conditioned medium from LPS-activated trout macrophages stimulated follicular contraction, suggesting that this process could be regulated by factors produced by activated immune cells. Collectively, these results indicate that in trout, ovarian steroid production, oocyte maturation and follicular contraction were not inhibited by an acute treatment with high doses of LPS *in vivo*. This lack of inhibitory effects of LPS support the notion that fish are remarkably resistant to LPS, a fact further corroborated by the lack of mortality during the study. However, since ovarian apoptosis was increased in response to LPS in the present study, it is possible that the number of viable ovarian follicular cells could progressively decrease with time, eventually causing alterations in normal ovarian function. Notably, under the present experimental conditions, female trout were sacrificed one day after the last LPS injection, and therefore, their ovarian function was evaluated right after the termination of the acute LPS treatment. Thus, future studies in our laboratory will assess the long-term effects of treatment with LPS on ovarian function, gamete production and viability.

In the current study, LPS administration elevated cortisol concentration, a result previously shown in several teleost species [66-69]. Since elevated cortisol concentration in the blood of female trout has been correlated with reproductive alterations, namely decreased plasma steroid concentration, delayed ovulation, reduced egg size and decreased survival in the progeny [70,71], we cannot rule out the possibility that cortisol may have contributed, at least in part, to the observed effects of LPS on apoptosis in the trout ovary. However, several of our findings would argue against this possibility. First, acute stressed-induced cortisol inhibits the production of ovarian steroids in trout [70]. However, despite a five-fold increase in cortisol concentration, the acute administration of LPS did not alter the production of ovarian steroids or the process of oocyte maturation in the present study. Second, cortisol has anti-apoptotic functions in fish and, therefore, if it were involved in the regulation of ovarian apoptosis it would be expected to antagonize the effects of LPS in the ovary. Instead, cortisol appears to modulate the response of *in vitro *differentiated trout macrophages to LPS [46]. Therefore, it is unlikely that elevated cortisol concentration is involved in the increased apoptosis in the ovary of LPS-injected females. It is also possible that cortisol may have had some anti-inflammatory actions in the trout ovary, as suggested by the decreased expression of two immune-related genes. The expression of the allograft inflammatory factor 1 (AIF-1), known to be involved in the immune response during macrophage activation and which is inhibited by the synthetic glucocorticoid dexamethasone [72], was decreased in the trout ovary in response to LPS. Further, the expression of the high affinity IgE receptor alpha subunit, known to be involved in IgE-induced allergen presentation in antigen-presenting cells and also inhibited by dexamethasone in mammals [73], was also decreased in response to LPS.

In conclusion, to our knowledge, we report for the first time the effects of LPS administration on ovarian function in fish. Our results indicate that an acute administration of LPS had no inhibitory effects on ovarian steroidogenesis, oocyte maturation or follicle contraction, but it caused a marked increase in apoptosis in the follicular layers surrounding the oocyte. At present, we do not know if the observed increase in LPS-induced apoptosis could cause changes in the number of follicular cells and/or alterations in ovarian function in fish. Future studies in our laboratory will be conducted to answer these questions.

## Authors' contributions

SM participated in the design of the study, in the execution of the experiments and in the writing of the manuscript. NM performed the TUNEL assays and participated in the generation of the sex steroid data. MM participated in the measurement of sex steroids. LA performed the cortisol assays. LT participated in the writing of the manuscript. AK performed the microarray analyses and participated in the writing of the manuscript. FWG participated in the design of the study, in the execution of the experiments and in the writing of the manuscript. JVP coordinated and participated in the design of the study as well as in the execution of the experiments and drafted the manuscript. All authors read and approved the final manuscript.
